# Effect of TDP2 on the Level of TOP2-DNA Complexes and SUMOylated TOP2-DNA Complexes

**DOI:** 10.3390/ijms19072056

**Published:** 2018-07-14

**Authors:** Ka Cheong Lee, Rebecca L. Swan, Zbyslaw Sondka, Kay Padget, Ian G. Cowell, Caroline A. Austin

**Affiliations:** 1 Institute for Cell and Molecular Biosciences, Newcastle University, Newcastle upon Tyne NE2 4HH, UK; larrylee585@gmail.com (K.C.L.); r.bramley@ncl.ac.uk (R.L.S.); z.sondka@gmail.com (Z.S.); ian.cowell@ncl.ac.uk (I.G.C.); 2 Department of Applied Biology, Northumbria University, Newcastle upon Tyne NE1 8ST, UK; kay.padget@northumbria.ac.uk

**Keywords:** topoisomerase II (TOP2), 5′-tyrosyl DNA phosphodiesterase 2 (TDP2), etoposide, proteasome, TOP2-DNA covalent complex, TARDIS, SUMO

## Abstract

DNA topoisomerase II (TOP2) activity involves a normally transient double-strand break intermediate in which the enzyme is coupled to DNA via a 5′-phosphotyrosyl bond. However, etoposide and other topoisomerase drugs poison the enzyme by stabilising this enzyme-bridged break, resulting in the accumulation of TOP2-DNA covalent complexes with cytotoxic consequences. The phosphotyrosyl diesterase TDP2 appears to be required for efficient repair of this unusual type of DNA damage and can remove 5′-tyrosine adducts from a double-stranded oligonucleotide substrate. Here, we adapt the trapped in agarose DNA immunostaining (TARDIS) assay to investigate the role of TDP2 in the removal of TOP2-DNA complexes in vitro and in cells. We report that TDP2 alone does not remove TOP2-DNA complexes from genomic DNA in vitro and that depletion of TDP2 in cells does not slow the removal of TOP2-DNA complexes. Thus, if TDP2 is involved in repairing TOP2 adducts, there must be one or more prior steps in which the protein-DNA complex is processed before TDP2 removes the remaining 5′ tyrosine DNA adducts. While this is partly achieved through the degradation of TOP2 adducts by the proteasome, a proteasome-independent mechanism has also been described involving the SUMOylation of TOP2 by the ZATT E3 SUMO ligase. The TARDIS assay was also adapted to measure the effect of TDP2 knockdown on levels of SUMOylated TOP2-DNA complexes, which together with levels of double strand breaks were unaffected in K562 cells following etoposide exposure and proteasomal inhibition.

## 1. Introduction

DNA topoisomerase II (TOP2) mediates important changes in DNA topology through the induction of a double strand DNA break (DSB), followed by the passage of another intact DNA molecule through the break. The ability of TOP2 enzymes to unwind and decatenate DNA is involved in many DNA-dependent processes including DNA replication, transcription, chromosome segregation and condensation. DSBs are lethal DNA lesions which remain concealed through the formation of a 5′phosphotyrosyl bond between the 5′ end of DNA and the TOP2 active site tyrosine. This covalent intermediate of the normal TOP2 reaction mechanism is termed the TOP2-DNA covalent complex. Following strand passage, the DSB is religated and TOP2 dissociates from DNA. However, a class of anticancer drugs called TOP2 poisons inhibit religation of the DSB, leading to the persistence of cytotoxic TOP2-DNA complexes and DSBs. TOP2 poisons such as etoposide, mitoxantrone, and doxorubicin are widely used in the clinic for the treatment of a range of malignancies but are also associated with the development of therapy-related leukaemia. TOP2 poison therapy could be improved through better understanding of how TOP2 poison–induced DNA damage is repaired.

Repair of TOP2 poison–induced DNA damage involves the processing of TOP2-DNA complexes to clean DNA ends which are required for religation. This can be achieved through multiple pathways, including nuclease-dependent mechanisms (involving, Mre11, CtIP or DNA2 [1,2,3,4,5,6]) and a proteolytic mechanism [7,8,9,10]. In the latter, the covalent TOP2 protein adduct is degraded by the proteasome, and remaining phosphotyrosyl peptides are removed through cleavage of the 5′-phosphotyrosine bond by TDP2.

TDP2 is a 5′-tyrosyl DNA phosphodiesterase which converts 5′-phosphotyrosine termini into 5′-phosphate termini in vitro [11,12]. Previously known as TTRAP or EAPII, TDP2 is a member of the divalent cation dependent phosphodiesterases, most closely related to APE1 [11]. While first identified as an ETS-interacting factor, it also modulates NF-kappa B activation, regulates left-right axis determination in zebrafish and has been shown to facilitate viral integration by HIV-1 integrase [13,14,15,16]. In addition to its 5′-phosphodiesterase activity, TDP2 can also remove 3′-tyrosine adducts in vitro with low efficiency, and in cells lacking the known 3′-tyrosyl DNA phosphodiesterase, TDP1 [11,17]. However, a specific role of TDP2 in the repair of TOP2 poison–induced DNA damage was demonstrated by RNAi-mediated knockdown of TDP2, which significantly increased sensitivity to etoposide but not the TOP1 inhibitor camptothecin or other DNA damaging agents like MMS [11]. The effect of TDP2 depletion on the sensitivity to etoposide has since been demonstrated in other studies using TDP2 knockout murine cells and chicken DT40 cells [12,18,19]. Consistently, etoposide-induced phosphorylation of histone H2AX, a frequently used biomarker of DSBs, is increased upon TDP2 knockout, indicating inhibition of DNA repair. Indeed, TDP2 generates ligatable ends required for the NHEJ repair of TOP2 poison–induced damage [18].

To date, TDP2 is the only known 5′-tyrosyl phosphodiesterase in mammalian cells [12]. However, how TDP2 mediates the repair of TOP2-DNA complexes remains unclear. TDP2 contains a narrow DNA binding pocket [20,21] which is insufficient to accommodate the large TOP2 adduct. Instead, studies have shown that prior digestion of TOP2 by the proteasome is required for the removal of TOP2 adducts from DNA. Specifically, TDP2 is able to cleave the 5′-phosphotyrosyl linkage between TOP2 and DNA only after tryptic digestion of the full TOP2 protein to smaller peptides [22], although TDP2 was able to remove larger 5′-tyrosyl peptides from DNA including digoxigenin adducts [23].

More recently, a proteasome-independent mechanism of removal by TDP2 was reported involving the SUMOylation of TOP2 by the ZATT SUMO ligase (previously known as ZNF451) [24,25], and ZNF451 was identified as a TOP2B-interacting protein in a biotin-ID interaction study [26]. TOP2-DNA complexes are SUMOylated in the presence of etoposide, which Schellenberg et al. show to be largely ZATT-dependent. This enhances the interaction of TOP2 with TDP2, which does not contain a canonical SUMO interacting motif (SIM) but does contain a number of SUMO binding elements. While the precise mechanism remains unclear, knockout of ZATT increases sensitivity to etoposide but not other DNA damaging agents, which is consistent with a specific role in the repair of TOP2 poison–induced DNA damage. It is important to note that the TDP2- and ZATT-dependent processing of TOP2-DNA complexes was only observed upon proteasome inhibition, supporting the notion that proteasomal removal of TOP2-DNA complexes is a major processing pathway.

Using our adapted trapped in agarose DNA immunostaining (TARDIS) assay [4], we tested whether active recombinant TDP2 protein is able to remove TOP2 from genomic DNA in vitro. Unlike 20S proteasomes, TDP2 alone was unable to remove TOP2 from genomic DNA. Furthermore, TDP2 depletion in cells did not affect the disappearance of etoposide-induced TOP2 complexes. This suggests that TDP2 represents an important end-polishing activity required to produce ligatable DNA-ends but that it does this in conjunction with other, probably proteolytic activities. Indeed, we show that the proteasome-independent processing of TOP2-DNA complexes by TDP2 does not contribute significantly to the removal of covalently bound TOP2 from DNA nor the appearance (or disappearance) of etoposide-induced DSBs.

## 2. Results

### 2.1. TDP2 Removes 5′-Tyrosine Adducts from a Labelled Oligonucleotide Substrate in Vitro

**Figure 1:**
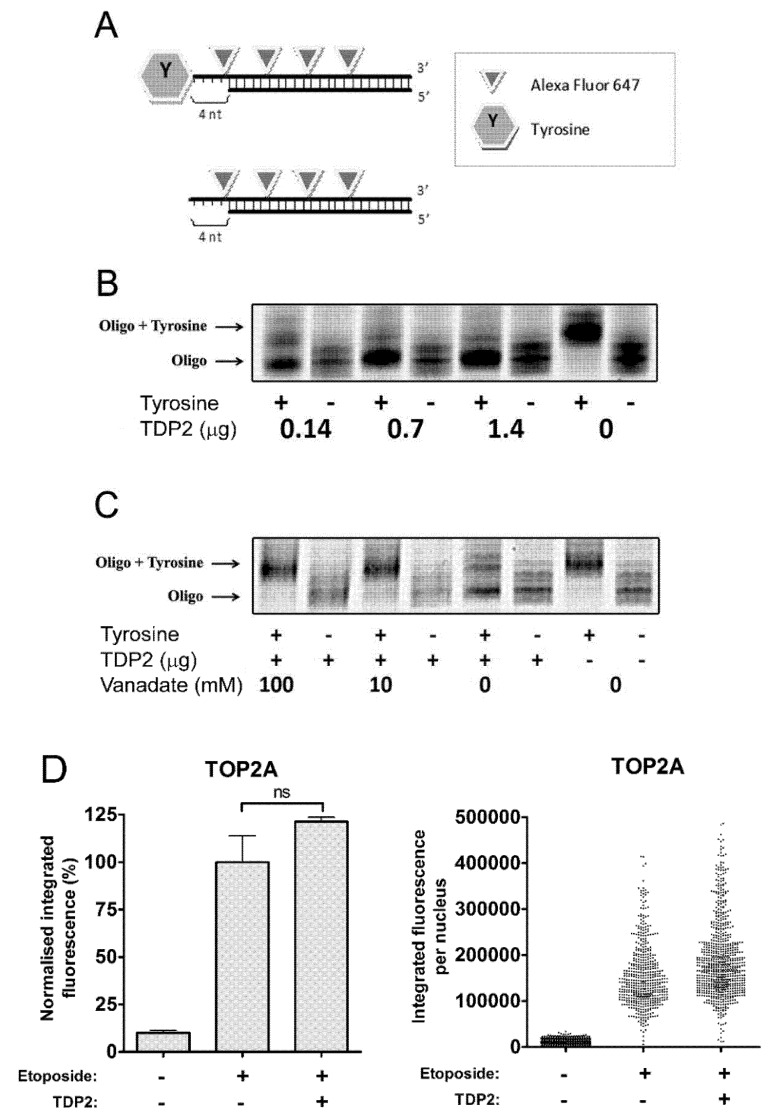
Effect of active TDP2 protein on the removal of topoisomerase II adducts in vitro. (**A**) Schematic of Alexa-647 labelled oligonucleotide substrate with or without tyrosine covalently attached to 5′-end. (**B**) Substrates were treated with 0.14, 0.7 or 1.4 μg of TDP2 (TTRAP) protein. (**C**) Oligonucleotide substrates were treated with 1.4 μg of TDP2 in the presence of 0, 10 or 100 mM vanadate. (**D**) Trapped in agarose DNA immunostaining (TARDIS) slides were prepared following treatment of K562 cells with 100 µM etoposide or DMSO for 2 h. After cell lysis, slides containing agarose-embedded genomic DNA bearing TOP2 adducts were incubated for 90 min with 1 µg active recombinant TDP2 (or TDP2 buffer). Levels of DNA-bound TOP2A and TOP2B remaining after incubation with recombinant protein were assessed by quantitative immunofluorescence (TARDIS analysis) [27]. All fluorescence values were normalised to the values obtained following exposure of cells to 100 µM etoposide, without subsequent treatment with TDP2. Scatter diagram shows raw integrated fluorescence values from a single experiment before normalisation. Statistical comparisons were made by unpaired *t*-test (*n* = 3).

TDP2 is a 5′-phosphodiesterase which directly cleaves the 5′-phosphotyrosyl bond between the TOP2 active site tyrosine and the phosphate backbone of DNA. The cleavage activity of recombinant TDP2 was verified using a double-stranded oligonucleotide substrate coupled to a tyrosine residue at the 5′ end (Figure 1A). Cleavage was detected by a change in electrophoretic mobility caused by loss of the 5′ tyrosine. Incubation of the oligonucleotide substrate with 0.14, 0.7 and 1.4 µg TDP2 protein resulted in the removal of the tyrosine residue from the 5′ end (Figure 1B). Furthermore, cleavage was inhibited in the presence of 10 mM or 100 mM vanadate, a known TDP2 inhibitor (Figure 1C) [12]. Therefore, the recombinant TDP2 protein used in the current study is an active 5′-phosphodiesterase which efficiently removes 5′-tyrosine adducts from an oligonucleotide substrate in vitro.

### 2.2. TDP2 Alone Does not Remove TOP2 Protein from Etoposide-Induced TOP2-DNA Covalent Complexes

We then used an adapted trapped in agarose immunostaining (TARDIS) assay [27] to determine whether TDP2 can remove etoposide-stabilised covalent TOP2 complexes from genomic DNA in vitro. Briefly, etoposide-treated and control cells were embedded in agarose on glass microscope slides and soluble cellular constituents were removed by SDS and salt extraction [27,28]. The resulting “ghosts” remaining in the agarose consist of genomic DNA and covalently linked TOP2 molecules that can be quantified by fluorescent microscopy. To assay the activities that might remove TOP2 from TOP2-DNA covalent complexes, slides bearing “ghosts” from etoposide-treated cells were incubated with catalytically active recombinant TDP2 protein and after washing, remaining TOP2 immunofluorescence was quantified. In a previous experiment to confirm that protein activities can penetrate the agarose and affect the DNA-bound TOP2, slides prepared from etoposide-treated cells were incubated with proteinase K. In this case, TOP2A fluorescence levels (corresponding to TOP2A-DNA covalent complexes) were reduced almost to background level, showing that the agarose does not pose a barrier to proteins reaching the nuclear ghosts. This is expected, as IgG antibody molecules are not hindered by the agarose in the immunofluorescence step of the procedure [4,29]. All subsequent analyses were carried out in the presence of protease inhibitors. While the recombinant TDP2 was active in the in vitro oligonucleotide assay, TDP2 did not remove TOP2A protein from genomic DNA (*p* = 0.1020, Figure 1D) or TOP2B (data not shown). Thus, TDP2 alone does not remove TOP2 protein adducts from DNA to generate clean ends for ligation in vitro.

**Figure 2:**
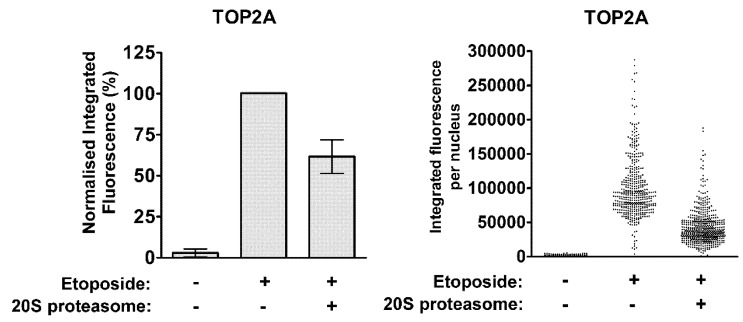
Incubation of TARDIS slides with 20S proteasomes. TARDIS slides prepared from etoposide-treated K562 cells were treated with 1 µg 20S proteasome preparations. After 90 min, remaining TOP2A-DNA covalent complexes were detected by quantitative immunofluorescence. All fluorescence values were normalised to the values obtained following exposure of cells to 100 µM etoposide and subsequent incubation in preparation buffer without 20S proteasomes. Data is presented as the normalised mean of medians ± SEM (histogram) and raw integrated fluorescence values from a single experiment before normalisation (scatter diagram).

In contrast, 20S proteasomes were able to remove TOP2A adducts from genomic DNA in a positive control experiment using the modified TARDIS assay (Figure 2). Untreated or etoposide-treated cells were prepared on TARDIS slides as above, followed by incubation of slides with 1 µg purified 20S proteasomes. Alternatively, TARDIS slides were incubated with preparation buffer without purified proteasomes. Levels of TOP2A-DNA complexes were reduced by approximately 40% following incubation with 20S proteasomes compared to buffer alone. This is consistent with the well-established role of the proteasome in the processing of TOP2-DNA complexes [7,8,9,10,30], and demonstrates the ability of proteins to penetrate the agarose and affect levels of DNA-bound TOP2 on TARDIS slides. Thus, the inability of TDP2 protein to remove TOP2 adducts from genomic DNA is not simply due to the inaccessibility of TOP2-DNA complexes once embedded in agarose.

### 2.3. TDP2 Depletion Does not Affect the Removal of Etoposide-Induced TOP2-DNA Covalent Complexes in Cells

**Figure 3:**
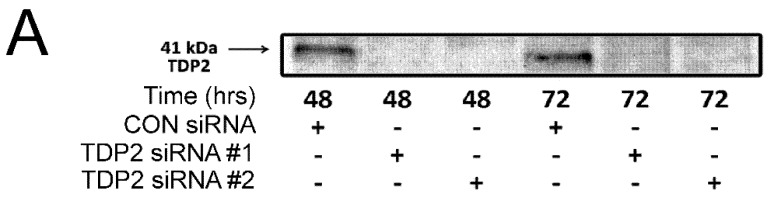

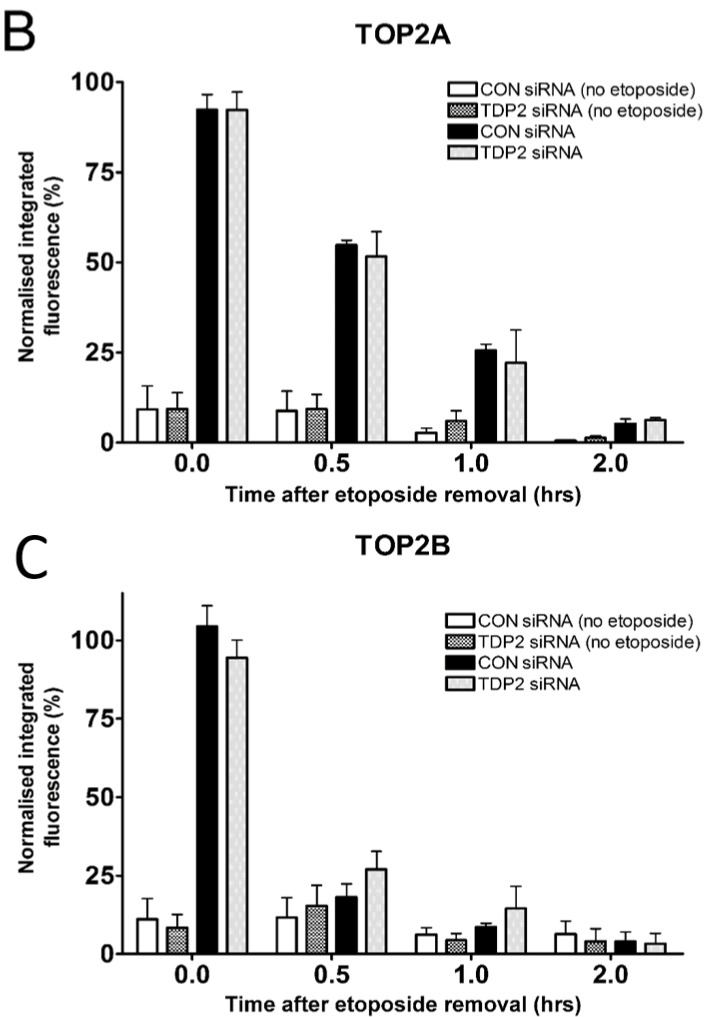
Cellular depletion of TDP2 does not affect topoisomerase II protein complex levels. TDP2 was depleted by two independent siRNAs. (**A**) Western blot visualising TDP2 protein levels after 48 and 72 h. (**B**,**C**) Cells transfected with 500 nM TDP2 siRNA #1 or non-coding (CON) siRNA were exposed to etoposide or solvent for 2 h and then placed in drug free media. TOP2A- and TOP2B-DNA complexes were quantified by TARDIS assay immediately after drug treatment and after 0.5, 1 and 2 h incubation in drug free media. Values are normalised to a 2 h 100 µM etoposide control (*n* = 6).

To determine whether TDP2 is required to resolve TOP2-DNA complexes within cells, TDP2 was depleted by siRNA. Two independent siRNAs were used and both depleted TDP2 protein levels at 48 and 72 h (Figure 3A). The effect of TDP2 knockdown on levels of TOP2A- and TOP2B- DNA complexes was then determined using the TARDIS assay. The level of TOP2 complexes present before the addition of etoposide was not affected by TDP2 depletion. Control and TDP2-depleted cells were treated with etoposide for 2 h, then harvested or placed in drug free media for 0.5, 1 or 2 h. In control cells, 50% of TOP2A-DNA complexes remained after 30 min of etoposide removal, as expected [30,31]. Consistent with previous studies [9,10,30,32], TOP2B-DNA complexes are removed more rapidly than TOP2A-DNA complexes, with less than 25% of TOP2B-DNA complexes remaining after 30 min of etoposide removal. Levels of TOP2A- and TOP2B-DNA complexes were not significantly affected by depletion of TDP2 at any of the time points tested (Figure 3B,C), suggesting that TDP2 alone does not remove etoposide-induced TOP2-DNA complexes.

### 2.4. The Proteasome-Independent Removal of TOP2-DNA Complexes by TDP2 is not Detectable in K562 Cells Following TDP2 Knockdown

**Figure 4:**
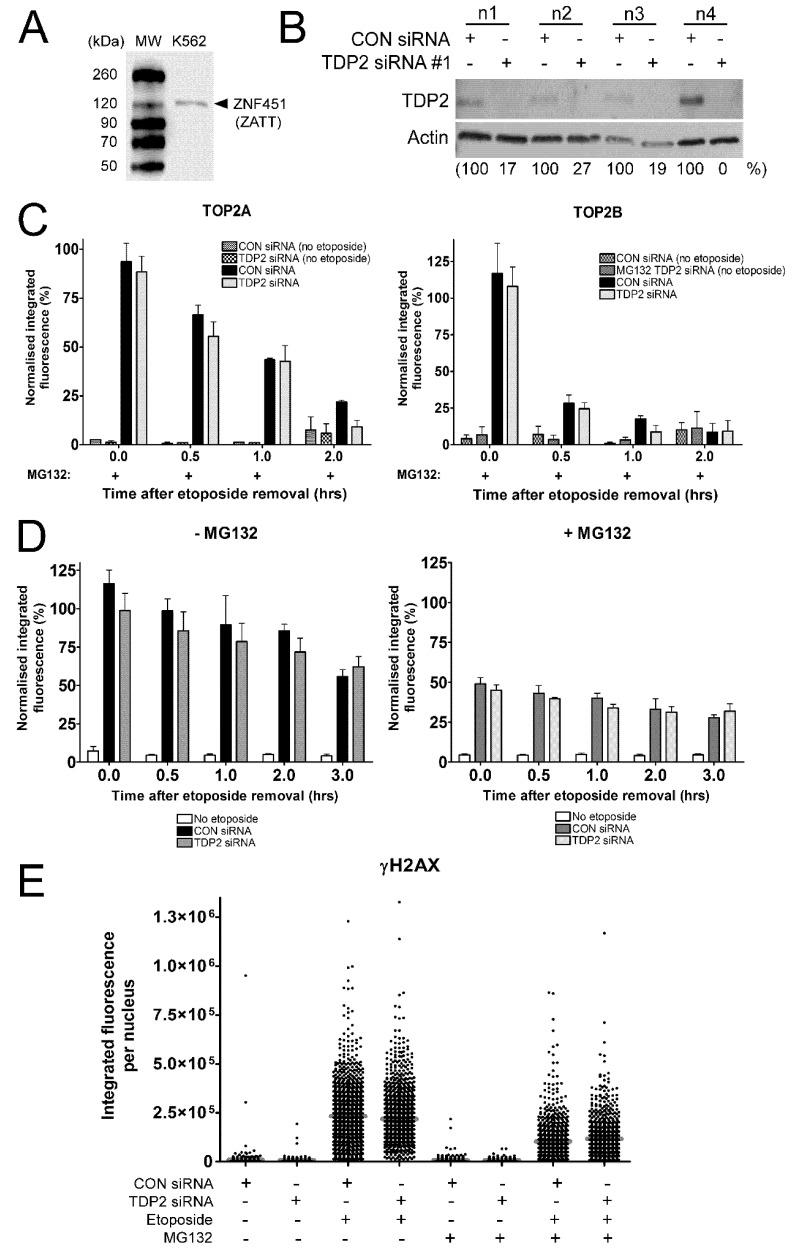
Effect of TDP2 siRNA knockdown on levels of etoposide-induced TOP2-DNA complexes and histone H2AX phosphorylation following proteasomal inhibition. (**A**) Expression of the ZATT SUMO ligase (ZNF451) in K562 cells, shown by western blot. (**B**) siRNA-mediated knockdown of TDP2 in K562 cells from four biological replicates from experiments shown in Figure 4B,C. Cells were transfected with 500 nM TDP2 siRNA #1 or non-coding (CON) siRNA and used for TARDIS and ɣH2AX assay 72 h after electroporation. (**C**) Control or TDP2 siRNA knockdown cells were treated with 100 µM etoposide and 10 µM MG132 for 2 h, followed by incubation in etoposide-free medium containing MG132 for 0, 0.5, 1 or 2 h. Levels of TOP2A- and TOP2B- DNA complexes were measured using the TARDIS assay. Values are normalised to a 2 h 100 µM etoposide control and compared by two-way ANOVA (*n* = 4). (**D**) Control or TDP2 siRNA knockdown cells were treated with 100 µM etoposide alone or in combination with 10 µM MG132 for 2 h, followed by incubation in etoposide-free medium containing solvent or MG132 for 0, 0.5, 1, 2, or 3 h. Level of etoposide-induced histone H2AX phosphorylation was measured using the ɣH2AX assay. Values are normalised to a 2 h 100 µM etoposide control and compared by two-way ANOVA (*n* = 3). (**E**) Data from Figure 4D is presented as a scatter plot of raw integrated fluorescence values of ɣH2AX signal in individual cells (*n* = 1). Data is shown for CON siRNA and TDP2 siRNA knockdown cells treated continuously with 100 µM etoposide (or DMSO) for 2 h, alone or in combination with 10 µM MG132 as indicated.

It was recently shown that TDP2 removes TOP2A- and TOP2B-DNA complexes in a SUMO-dependent manner when the proteasome is inhibited [24]. The proteasome-independent removal of TOP2-DNA complexes by TDP2 involves the ZATT SUMO E3 ligase, which is expressed in K562 cells (Figure 4A). To investigate this TDP2-dependent pathway, the TARDIS assay was performed in control and TDP2 knockdown cells in the presence of proteasome inhibitor MG132. Figure 4B shows the level of TDP2 protein in four biological replicates for use in the TARDIS assay. Control or TDP2-depleted K562 cells were treated with 100 µM etoposide and 10 µM MG132 for 2 h, followed by incubation in etoposide-free medium containing MG132 for 0, 0.5, 1, or 2 h. Co-treatment of cells with MG132 slowed the removal of etoposide-induced TOP2-DNA complexes, with approximately 75% of TOP2A-DNA complexes remaining 0.5 h after etoposide removal as previously shown (Figure 4C) [30]. However, levels of TOP2A- and TOP2B- DNA complexes were unaffected by TDP2 depletion in MG132-treated K562 cells, and this was true at all time points tested.

The proteasome-independent removal of TOP2-DNA complexes by TDP2 was also investigated using the ɣH2AX assay. Removal of TOP2 from the TOP2-DNA complex is required for the liberation of the TOP2-mediated double strand DNA break (DSB) and subsequent activation of the DNA damage response [8,9,10,33]. Phosphorylation of histone H2AX at serine 139 (ɣH2AX) occurs upon recognition of DSBs by DNA damage-dependent kinases ATM, ATR, and DNA-PK and is one of the earliest markers of DSBs [34,35,36]. This can be detected using an anti-phospho-histone H2AX antibody and quantified by immunofluorescence. To investigate the role of TDP2 in the proteasome-independent processing of TOP2-DNA complexes to protein-free DSBs, control or TDP2 knockdown cells were treated with 100 µM etoposide alone or in combination with 10 µM MG132 for 2 h. Cells were then incubated in etoposide-free medium containing solvent or MG132 for 0, 0.5, 1, 2, or 3 h, and levels of phosphorylated histone H2AX were quantified by ɣH2AX assay.

As expected, the level of etoposide-induced ɣH2AX signal was significantly reduced in the presence of MG132, reflecting the role of the proteasome in the removal of TOP2-DNA complexes and processing to DSBs (Figure 4D,E) [8,10]. Consistent with Figure 2, depletion of TDP2 did not affect the appearance of etoposide-induced DSBs in the absence of MG132, suggesting that TDP2 alone cannot remove covalently bound TOP2 from DNA. However, the average levels of etoposide-induced DSBs (as well as the distribution of the response in the cell population, shown in Figure 4E) were also unaffected by TDP2 depletion when the proteasome was inhibited (Figure 4D). This suggests that TDP2 is not required for the appearance of etoposide-induced DSBs when the proteasomes are inhibited.

Therefore, proteasome-independent removal of TOP2-DNA complexes by TDP2 was not observed in K562 cells following TDP2 depletion by TARDIS assay or ɣH2AX assay. This is in contrast to the findings presented by Schellenberg et al., who show that the number of etoposide-induced ɣH2AX foci and levels of TOP2-DNA complexes (measured by ICE assay) are substantially increased in TDP2 knockout cells in the presence of MG132. An important difference in the aforementioned study is the use of TDP2 knockout cells which contain no detectable TDP2 protein [24]. It is plausible that remaining TDP2 protein in siRNA-treated cells may contribute to the removal of TOP2-DNA complexes. Nonetheless, this suggests that the proteasome-independent removal of TOP2-DNA complexes by TDP2 is not a major removal pathway in K562 cells.

### 2.5. Investigating the Proteasome-Independent Removal of SUMOylated TOP2-DNA Complexes in TDP2 Knockdown Cells Using the TARDIS Assay

**Figure 5:**
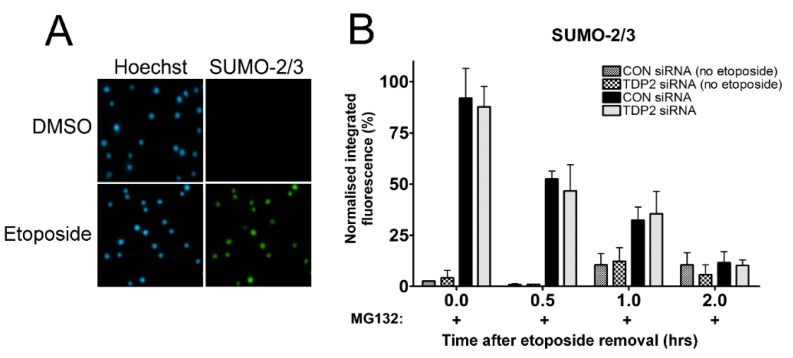
Effect of TDP2 siRNA knockdown on levels of SUMOylated TOP2-DNA complexes following proteasomal inhibition. (**A**) K562 cells were treated with 100 µM etoposide (or equivalent volume of DMSO) for 2 hours, and SUMOylated TOP2-DNA complexes were visualized using the TARDIS assay; (**B**) Control or TDP2 siRNA knockdown cells were treated with 100 µM etoposide and 10 µM MG132 for 2 h, followed by incubation in etoposide-free medium containing MG132 for 0, 0.5, 1, or 2 h. Levels of SUMOylated TOP2-DNA complexes were measured using the TARDIS assay. Values are normalised to a 2 h 100 µM etoposide control and compared by two-way ANOVA (*n* = 3).

It is possible that changes in the level of SUMOylated TOP2-DNA complexes targeted in the proteasome-independent TDP2 removal pathway are not detectable through measurement of total TOP2 complex levels. This may be true if only a small proportion of TOP2-DNA complexes are SUMOylated at one time, which is likely given the highly reversible nature of SUMO conjugation. Therefore, the TARDIS assay was adapted for the measurement of SUMOylated TOP2-DNA complexes by probing TARDIS slides with anti-SUMO antibodies. In the absence of TOP2 poison, stringent lysis in buffer containing SDS and high salt removes all SUMO-2/3 conjugates from agarose-embedded genomic DNA (Figure 5A). However, SUMO-2/3 is detected on slides following treatment of cells with etoposide, reflecting SUMOylated TOP2-DNA complexes (Figure 5A). Indeed, other potentially modified proteins which may be trapped or tightly associated with DNA following etoposide treatment (such as RNA polymerase II or Ku70/80, which are readily detectable by standard immunofluorescence) are also removed from TARDIS slides (unpublished data). This approach was therefore used to investigate the proteasome-independent removal of SUMOylated TOP2-DNA complexes in control and TDP2 depleted cells.

CON siRNA- or TDP2 siRNA-treated K562 cells shown in Figure 4A were incubated with 100 µM etoposide in combination with 10 µM MG132 for 2 h. Etoposide was removed from the culture medium, and cells were collected or incubated for a further 0.5, 1, or 2 h in medium containing 10 µM MG132 to maintain proteasome inhibition. As expected, levels of remaining SUMOylated TOP2-DNA complexes were reduced with time after etoposide removal, representative of proteasome-independent removal (Figure 5B) [30]. However, levels of remaining SUMOylated TOP2 complexes were unaffected by TDP2 knockdown, suggesting that TDP2 is not required for the proteasome-independent removal of SUMOylated TOP2-DNA complexes.

## 3. Discussion

TDP2 is involved in the repair of TOP2 poison–induced DNA damage. Specifically, TDP2 removes 5′-tyrosine adducts from DNA, including the 5′-phosphotyrosyl peptides which remain following partial digestion of TOP2-DNA complexes by the proteasome [11,12,22]. After the initial proteolytic cleavage of TOP2, TDP2 is required for the conversion of the remaining 5′-phosphotyrosine or phosphotyrosyl peptide to a 5′-phosphate, rendering the DNA end ligatable for NHEJ repair. It is generally accepted that additional factors are required for the removal of TOP2-DNA complexes by TDP2, including the proteasome and the ZATT SUMO E3 ligase (previously known as ZNF451). In support of this, we have shown that, unlike proteasome inhibition, depletion of TDP2 alone does not affect the removal of covalently bound TOP2 from DNA nor the appearance of etoposide-induced DSBs.

In addition, the proteasome–independent mechanism of TOP2-DNA complex removal by TDP2 was investigated [24], but was not observed in TDP2-depleted K562 cells following siRNA knockdown. TOP2-DNA complexes are SUMOylated in the presence of epipodophyllotoxins [36,37]. Only a small population of total protein is SUMOylated at one time due to the highly reversible nature of SUMO conjugation. It is therefore plausible that TDP2-dependent removal of SUMOylated TOP2-DNA complexes is not detectable through the measurement of total protein levels. However, TDP2-dependent removal was also undetectable when TARDIS slides were probed for SUMO-2/3 rather than total TOP2-DNA complexes. Furthermore, depletion of TDP2 did not affect the appearance or disappearance of etoposide-induced DSBs upon proteasomal inhibition. This suggests that the direct removal of SUMOylated TOP2-DNA complexes via a proteasomal independent mechanism (reported by Schellenberg et al. and mediated by the ZATT SUMO ligase) is not a major mechanism of TOP2-DNA complex removal in K562 cells.

Catalytically active TDP2 protein was unable to remove TOP2 from genomic DNA embedded in agarose. One concern is whether TDP2 can penetrate the agarose on slides to access TOP2-DNA covalent complexes. However, various studies have shown that proteins can enter agarose to access DNA. For example, *Fpg* treatment of agarose embedded cells is used to enhance the detection of oxidative DNA damage [37], DNaseI digestion, and subsequent linker ligation is carried out in agarose plugs in during DNase-seq [38], and we have shown that proteinase K and MRE11 can remove TOP2-DNA complexes from genomic DNA in TARDIS slides [4]. In the current study, we have also demonstrated that TOP2-DNA complexes are removed from TARDIS slides following incubation with 20S proteasome preparations. Therefore, the inability of TDP2 to remove TOP2 from TARDIS slides is not simply due to an inability to access agarose-embedded TOP2-DNA complexes.

Consistently, TDP2 depletion (unlike proteasome inhibition) did not affect the appearance of etoposide-induced DSBs, which is dependent on the removal of TOP2 protein from the TOP2-mediated DSB. Surprisingly, TDP2 depletion also did not affect the disappearance of etoposide-induced DSBs as reported by others in TDP2 knockout cells [18,39], which may reflect differences between TDP2 knockdown cells and TDP2 knockout cells. While TDP2 is the major or possibly only 5′-tyrosyl phosphodiesterase in vertebrate cells [12], it is probable that TDP2-independent pathways for processing TOP2-DNA complexes exist. In support of this, although chicken DT40 cells null for TDP2 are hypersensitive to etoposide in clonogenic survival assays, the sensitization appears to be considerably less than that observed previously for DT40 cells null for LIG4 or KU, which are central components of the NHEJ pathway [12,40]. TDP2-independent strategies employed by the cell may involve nucleolytic activities which may or may not depend on prior degradation of TOP2. In *Saccharomyces cerevisiae*, TOP2 and TOP2-like SPO11 protein-DNA adducts are removed endonucleolytically in a process that involves the MRE11 and CtIP homologues Rad32 and Ctp1 [41]. In addition, we have shown that immunopurified or recombinant MRE11 can remove TOP2 complexes from genomic DNA in vitro and that endogenous levels of TOP2-DNA covalent complexes are elevated in cells genetically deficient for MRE11 nuclease or exposed to an MRE11 nuclease inhibitor [4]. Ku70/80 is an AP lyase which is also implicated in the processing of TOP2 poison–induced DNA damage as siRNA knockdown of Ku70 increases etoposide sensitivity independently of NHEJ repair [5,42]. Therefore, the processing of TOP2 poison–induced DNA damage involves a number of repair pathways, which may predominate according to how and when the DNA damage occurs.

Understanding the pathways for processing of TOP2-DNA complexes has implications for the use of TOP2 poisons, both in the identification of useful chemotherapeutic targets and potentially in minimising damaging chromosomal translocations that are associated with TOP2 poisons. The data presented here supports a model whereby TDP2 is required for the “end-polishing” steps of TOP2-DNA complex repair. Other activities are required for the removal of full length TOP2 protein from TOP2-DNA complexes, such as proteasomal degradation.

## 4. Materials and Methods

### 4.1. TDP2 Activity Assay

Oligonucleotide Tyr with a tyrosine residue coupled to the 5′-end was synthesized by Eurogentec (Tyr; Tyr-GCGCTGACAAGCTGAGGATCGTCATCCTCG, Tyr_complement; CGAGGATCGAGGATGACGATCCTCAGCTTGTCA) and labelled with AlexaFluor 647 with the Ulysis Nucleic Acid Labeling Kit (Molecular Probes). To simulate TOP2 cleavage products, primers Tyr and Tyr_complement were annealed by heating to 95 °C for 2 min in annealing buffer (10 mM Tris, pH 7.5–8.0, 50 mM NaCl, 1 mM EDTA) followed by cooling to room temperature over 1 h to form a 26 bp double-strand DNA oligomer with 4 base 5′-overhang with tyrosine attached to the 5′-end. A construct with identical primers lacking the tyrosine was used as a control (Tyr_MINUS; CGCTGACAAGCTGAGGATCGTCATCCTCG, Eurogentec).

Double-stranded oligonucleotides were used as substrates in reaction with 0.14, 0.7 or 1.4 μg of recombinant TDP2 protein (residues 1-363 with an N-terminal GST tag, Abnova, Taipei, Taiwan). Reactions were performed at 37 °C for 30 min. Reaction products were denatured in formamide buffer (20 mM EDTA, 40 mM NaOH in formamide) containing unlabelled competitor DNA oligonucleotide (Alexa_competitor; GCGCTGACAAGCTGAGGATCGTCATCCTCG, Eurogentec, Seraing, Belgium) for “Tyr” primer and analyzed on a sequencing polyacrylamide gel (15% PA, 7 M urea) where a difference in mobility caused by the presence/absence of the tyrosine moiety was observed. Results were visualised using a Typhoon Phosphorimager (GE Healthcare, Chicago, IL, USA). In this study, 1.4 μg of recombinant TDP2 protein was used to remove the tyrosine residue from 5′-end of DNA oligonucleotide in the presence of 10 or 100 mM vanadate. Reaction products were denatured in formamide buffer containing unlabelled competitor DNA oligonucleotide for “Tyr” primer and analyzed as above.

### 4.2. In Vitro Trapped in Agarose Immunostaining (TARDIS)

TOP2 adducts on genomic DNA were generated by treating K562 cells with 100 µM etoposide for 2 h prior to embedding cells in agarose on microscope slides as described before [27,43]. Active TDP2 (140 µg/mL) was diluted in NEbuffer 4 (New England Biolabs, Ipswich, MA, USA) and applied to microscope slides bearing TOP2 protein adducts for 90 min at 37 °C. Alternatively, TARDIS slides were incubated with 1 µg of 20S proteasome preparation (obtained from Enzo Life Sciences, Farmingdale, NY, USA, Cat# 8720), and control slides were incubated in parallel with buffer alone. Slides were then washed and incubated with anti-TOP2A or anti-TOP2B rabbit polyclonal antibody (18511 or 18513, respectively) diluted 1:50 in 1% PBS + 0.1% Tween. After this, 1.5 h the antibody was removed, and the slides washed prior to incubation with FITC labelled anti-rabbit IgG (Sigma, St. Louis, MO, USA, F1262 s antibody diluted 1:1000 in 1% PBS + 0.1% Tween. In experiments utilizing TDP2 siRNA knockdown, TDP2 depleted and control cells were treated with 100 µM etoposide (alone or in combination with 10 µM MG132) for 2 h, and then cells were suspended in etoposide-free medium containing no drug, solvent, or 10 µM MG132 as indicated. Cells were collected at 0, 0.5, 1, and 2 h after drug removal, and TOP2A and TOP2B complexes were quantified by TARDIS analysis as previously described [27] using rabbit polyclonal antibodies 18511α and 18513β or 4566 and 4555.

### 4.3. TDP2 siRNA

TDP2 siRNA #1 (Hs_TTRAP4) and TDP2 siRNA #2 (Hs_TTRAP5) were obtained from Qiagen, Hilden, Germany with sequences 5′-CTGAAGATACTCAGCAAGAAA-3′ and 5′-CGGAACGAAYGAATCAGTTAA-3′, respectively. K562 cells were electroporated in 200 μL culture medium at a density of 2 × 10^7^ cells/ml in 4 mm electroporation cuvettes. siRNAs were added at a concentration of 500 nM immediately before electroporation. Electroporations were performed using a rectangle pulse EPI 2500 electroporator (Fischer, Heidelberg, Germany). After 15 min recovery at room temperature, cells were diluted into fresh medium and incubated at 37 °C in 5% CO_2_. The efficiency of TDP2 knockdown was assessed by Western blotting.

### 4.4. Western Blotting

Cells were collected at 48 and 72 h after electroporation and whole cell extracts were prepared. Equivalent loadings (of approximately 1 × 10^5^ cells) were applied to each lane. TDP2 was detected with rabbit anti-TDP2 antibody (Abcam, Cambridge, UK, ab33246), and ZATT (ZNF451) was detected with rabbit anti-ZNF451 (Sigma, SAB2108741) The WesternSure pre-stained chemiluminescent protein ladder was purchased from LiCor, Lincoln, NE, USA (#926-98000).

## Abbreviations

ATM	Ataxia telangiectasia mutated kinase
ATR	Ataxia telangiectasia and Rad3 related kinase
ɣH2AX	Phosphorylated histone H2AX (ser 139)
DMSO	Dimethyl sulfoxide
DNA	Deoxyribonucleic acid
DNA-PK	DNA-dependent protein kinase
DSB	Double strand DNA break
FITC	Fluorescein isothiocyanate
NaOH	Sodium hydroxide
NHEJ	Non-homologous end joining
PBS	Phosphate buffered saline
SDS	Sodium dodecyl sulfate
SIM	SUMO interacting motif
siRNA	Small interfering RNA
SUMO	Small ubiquitin-like modifier
TOP2	DNA Topoisomerase II
TDP2	5′-tyrosyl DNA phosphodiesterase 2
TARDIS	Trapped in agarose DNA immunostaining
ZATT	Zinc finger protein associated with TDP2 and TOP2
